# t-Darpp Promotes Enhanced EGFR Activation and New Drug Synergies in Her2-Positive Breast Cancer Cells

**DOI:** 10.1371/journal.pone.0132267

**Published:** 2015-06-29

**Authors:** Erin C. Denny, Susan E. Kane

**Affiliations:** Department of Cancer Biology, Beckman Research Institute at City of Hope, Duarte, California, United States of America; University of South Alabama, UNITED STATES

## Abstract

Trastuzumab has led to improved survival rates of HER2^+^ breast cancer patients. However, acquired resistance remains a problem in the majority of cases. t-Darpp is over-expressed in trastuzumab-resistant cell lines and its over-expression is sufficient for conferring the resistance phenotype. Although its mechanism of action is unknown, t-Darpp has been shown to increase cellular proliferation and inhibit apoptosis. We have reported that trastuzumab-resistant BT.Her^R^ cells that over-express endogenous t-Darpp are sensitized to EGFR inhibition in the presence (but not the absence) of trastuzumab. The purpose of the current study was to determine if t-Darpp might modulate sensitivity to EGFR inhibitors in trastuzumab-resistant cells. Using EGFR tyrosine kinase inhibitors AG1478, gefitinib and erlotinib, we found that trastuzumab-resistant SK.Her^R^ cells were sensitized to EGFR inhibition, compared to SK-Br-3 controls, even in the absence of trastuzumab. t-Darpp knock-down in SK.Her^R^ cells reversed their sensitivity to EGFR inhibition. Increased EGFR sensitivity was also noted in SK.tDp cells that stably over-express t-Darpp. High levels of synergy between trastuzumab and the EGFR inhibitors were observed in all cell lines with high t-Darpp expression. These cells also demonstrated more robust activation of EGFR signaling and showed greater EGFR stability than parental cells. The T75A phosphorylation mutant of t-Darpp did not confer sensitivity to EGFR inhibition nor activation of EGFR signaling. The over-expression of t-Darpp might facilitate enhanced EGFR signaling as part of the trastuzumab resistance phenotype. This study suggests that the presence of t-Darpp in HER2^+^ cancers might predict the enhanced response to dual HER2/EGFR targeting.

## Introduction

Breast cancer represents the most common cancer in women worldwide with an estimated 1.6 million new cases diagnosed each year [1, 2]. Approximately 25–30% of these women present with an over-expression of human epidermal growth factor receptor 2 (HER2) [3]. The amplification of HER2, a receptor tyrosine kinase encoded by the ERBB2 oncogene, correlates with a poor prognosis and a poor response to chemotherapy [4]. Trastuzumab, a humanized monoclonal antibody targeting the extracellular region of HER2, remains the primary treatment for HER2^+^ breast cancer patients. Despite the specificity and efficacy of trastuzumab, trastuzumab monotherapy is only effective in about 30–45% of patients. Response rates are improved by the addition of chemotherapy to the treatment regimen, but approximately 75% of patients treated with trastuzumab will still develop resistance within one year [5, 6]. Although the mechanism of resistance is still largely unknown, *in vitro* and *in vivo* data have confirmed that sustained signaling through the PI3K/Akt signaling pathway and phosphorylation of Akt are largely responsible for the resistance phenotype [7, 8].

One potential mechanism for sustained downstream signaling in the presence of trastuzumab is by compensatory signaling using a different HER family receptor, such as EGFR or HER3. Co-expression of EGFR occurs in 35–65% of HER2^+^ breast cancer and is associated with a worse clinical prognosis than for breast cancers that don’t express EGFR [9–12]. We have previously shown that trastuzumab-resistant BT.Her^R^ cells are more sensitive to an EGFR tyrosine kinase inhibitor (TKI) in the presence of trastuzumab than in its absence, suggesting that those cells gain a dependence on EGFR when HER2 signaling is shut down [13]. More recent work has demonstrated that EGFR inhibitors are synergistic with trastuzumab in models of HER2^+^ breast cancer [14–16], again suggesting that EGFR is important as an alternative pathway when HER2 is inhibited.

We and others have reported that upregulation of the *PPP1R1B* gene plays a role in the trastuzumab resistance mechanism [17–20]. *PPP1R1B* codes for the 32kDa dopamine and cAMP-regulated phosphoprotein, Darpp-32, and its amino-truncated isoform, t-Darpp. Although Darpp-32 has been well characterized in neuronal cells as a dual-function phosphoprotein that inhibits protein kinase A (PKA) and protein phosphatase-1 (PP-1), its role in cancer has only been studied more recently [21, 22]. t-Darpp is frequently over-expressed in human adenocarcinomas of the esophagus, prostate, stomach, colon and breast [23] and over-expression of t-Darpp is sufficient to confer resistance to trastuzumab in HER2^+^ breast cancer [17, 18, 24]. Although the mechanism by which this occurs remains unclear, several groups have shown that t-Darpp upregulates cell growth through activation of the PI3K/Akt pathway and increased anti-apoptotic response through upregulation of Bcl-2 [18, 19, 24]. In this study, we examine the possible role of t-Darpp in conferring trastuzumab resistance via an effect on EGFR signaling. Our results suggest a novel function for t-Darpp by sensitizing breast cancer cells to EGFR inhibition.

## Materials and Methods

### Cell culture and reagents

The human breast cancer cell lines BT474 and SK-Br-3 were obtained from the American Type Culture Collection (Rockville, MD). BT.Her^R^ cells were generated as previously described through selection in the presence of trastuzumab [13]. BT474 cells and BT.Her^R^ cells were maintained in DMEM with 10% FBS and 1% penicillin/streptomycin. SK.Her^R^ cells were also generated through selection in the continuous presence of trastuzumab and were a kind gift from Rita Nahta [25]. Western hybridizations showing the relative levels of t-Darpp and Darpp-32 in parental and resistant BT474 and SK-Br-3 cells are shown in S1 Fig. Stably transfected SK-Br-3 cells expressing pcDNA3 empty vector (SK.empty), t-Darpp (SK.tDp), or t-Darpp plus flag-Darpp-32 (SK.dDp) were described previously [17]. SK-Br-3 cells were also stably transfected with a phosphorylation mutant of t-Darpp in which threonine 75 (T75) was mutated to an alanine (SK.tDp-T75A). SK-Br-3 cells, SK.Her^R^ and the stably transfected clones were maintained in McCoys 5A media with 10% FBS, 1% penicillin/streptomycin and 1% L-glutamine. SK.empty, SK.tDp and SK.tDp-T75A cells were maintained in media containing 500 μg/ml G418. SK.dDp cells were maintained in media containing 500 μg/ml G418 and 200 μg/ml zeocin. Trastuzumab was purchased from the City of Hope Hospital Pharmacy. AG1478 (Calbiochem, Ballerica, MA), gefitinib and erlotinib (LC Laboratories, Woburn, MA) were dissolved in DMSO and further diluted with cell culture media to concentrations described in the figure legends. p-EGFR (Y1068) (#2234), EGFR (#2232) were obtained from Cell Signaling (Danvers, MA). H62, an antibody that recognizes both Darpp-32 and t-Darpp, was obtained from Santa Cruz Biotechnology (Santa Cruz, CA, sc-11365) and β-actin (A4700) and α-tubulin (T5168) antibodies were obtained from Sigma-Aldrich Corporation (St. Louis, MO).

### Sulforhodamine B (SRB) assay

SRB assays were performed as previously described [13]. Briefly, cells were plated at a density of 4x10^3^ per well in 96 well plates and allowed to attach overnight. On day 0, media containing 0.1% DMSO or increasing concentrations of EGFR inhibitor (AG1478, gefitinib or erlotinib) were added to the cells. After 5 or 7 days for SK-Br-3 or BT474 cells, respectively, cells were fixed with 10% cold trichloroacetic acid (Sigma) and stained with 0.4% SRB in 1% acetic acid. The SRB stain was released using 10mM Tris base. Absorbances at 565 nm and 695 nm were read using a spectrophotometer. IC_50_ values were determined using CalcuSyn. Each experiment was run in quadruplicate and repeated a minimum of three times.

### siRNA gene knock-down

Cells were plated at a density of 2x10^5^ cells per well in 6-well plates. After attaching overnight, cells were transfected with scrambled siRNA or t-Darpp siRNA (Santa Cruz Biotechnology) using Lipofectamine 2000 (Invitrogen by Life Technologies, Grand Island, NY). After 24 hours, media was changed and treatments were carried out as described in figure legends.

### Western analysis

Cells were lysed using a modified RIPA buffer with 1mM Na_3_VO_4_ and a complete tablet of protease inhibitors (Roche, Indianapolis, IN). Protein concentrations were determined using the Bio-Rad Protein Assay (Bio-Rad, Hercules, CA). Proteins were separated on a 10 or 12% SDS-polyacrylamide gel or a precast 4–12% gradient NuPAGE Bis-Tris gel (Life Technologies) and transferred onto a nitrocellulose membrane. Membranes were probed with specific primary antibodies followed by horseradish peroxidase-coupled secondary antibodies (Cell Signaling). Chemiluminescence was detected using an ECL Plus kit (Thermo Fisher Scientific, Waltham, MA).

### Drug combination analysis

Serial dilutions of a fixed 2:1 ratio of EGFR inhibitor (AG1478, gefitinib or erlotinib) and trastuzumab were used for the combination studies. Each drug was added alone and in combination for each experiment. Combination analysis was performed using the method of Chou and Talalay and the CalcuSyn software program [26]. Each experiment was run in quadruplicate and repeated three times. Combination index (C.I.) values of less than 0.9 are synergistic, 0.9–1.1 are additive, and greater than 1.1 are antagonistic.

### Cycloheximide protein stability assay

Cells were plated at a density of 3.5x10^5^ cells per well in 6-well plates and incubated with 80 μg/ml cycloheximide (Calbiochem) to inhibit new protein synthesis. Cells were harvested at 12, 24, 36 and 48 hours after the addition of cycloheximide. Protein lysates were collected and analyzed by Western hybridization as described above, probing for total EGFR. Protein bands were quantified using ImageJ software and the EGFR band intensities for each time point were normalized to α-tubulin. The protein degradation curve was produced by plotting the band intensity ratio (relative to time 0) as a function of cycloheximide treatment time. Protein half-life was calculated using the best-fit line and determining the time at which 50% of the original protein had degraded.

### Colony formation assay

Cells were plated at a density of 500 cells per well in 6-well plates and allowed to attach overnight. Cells were treated with 0.1% DMSO or increasing concentrations of AG1478. Media was changed twice per week. Colonies, defined as having at least 50 cells, were counted after 14 days.

### EGFR stimulation assay

Cells were plated at a density of 3.5x10^5^ cells per well in 6-well plates and allowed to attach overnight. Cells were then serum-starved overnight (16 hours), followed by EGF (100 ng/ml) stimulation for the indicated time periods at 37°C. Protein lysates were collected and analyzed by Western hybridization as described previously to probe for p-EGFR (Y1068), total EGFR and α-tubulin. Protein bands were quantified using ImageJ Software. p-EGFR levels were normalized to α-tubulin and the relative p-EGFR activation levels were calculated relative to the zero time point for the appropriate parental cells in each case.

### Statistical analysis

Statistical significance was calculated using the GraphPad Prism 6.0 statistical software. Differences between groups were determined by the two-tailed Student’s *t*-test or the one-way ANOVA with Dunnett’s post-test. *p* values less than 0.05 were considered significant.

## Results

### Sensitivity to EGFR inhibition in trastuzumab-resistant cell lines

We previously observed that trastuzumab-resistant BT474 (BT.Her^R^) cells are more sensitive to AG1478, an EGFR tyrosine kinase inhibitor, in the presence but not the absence of trastuzumab [13]. As a more rigorous determination of synergy, we used a combination index analysis to study the interaction of trastuzumab with EGFR-specific kinase inhibitors in parental and trastuzumab-resistant cell lines. The combination of either AG1478 or erlotinib with trastuzumab produced high levels of synergy in both the parental BT474 and the BT.Her^R^ cells (S1 Table).

**Fig 1:**
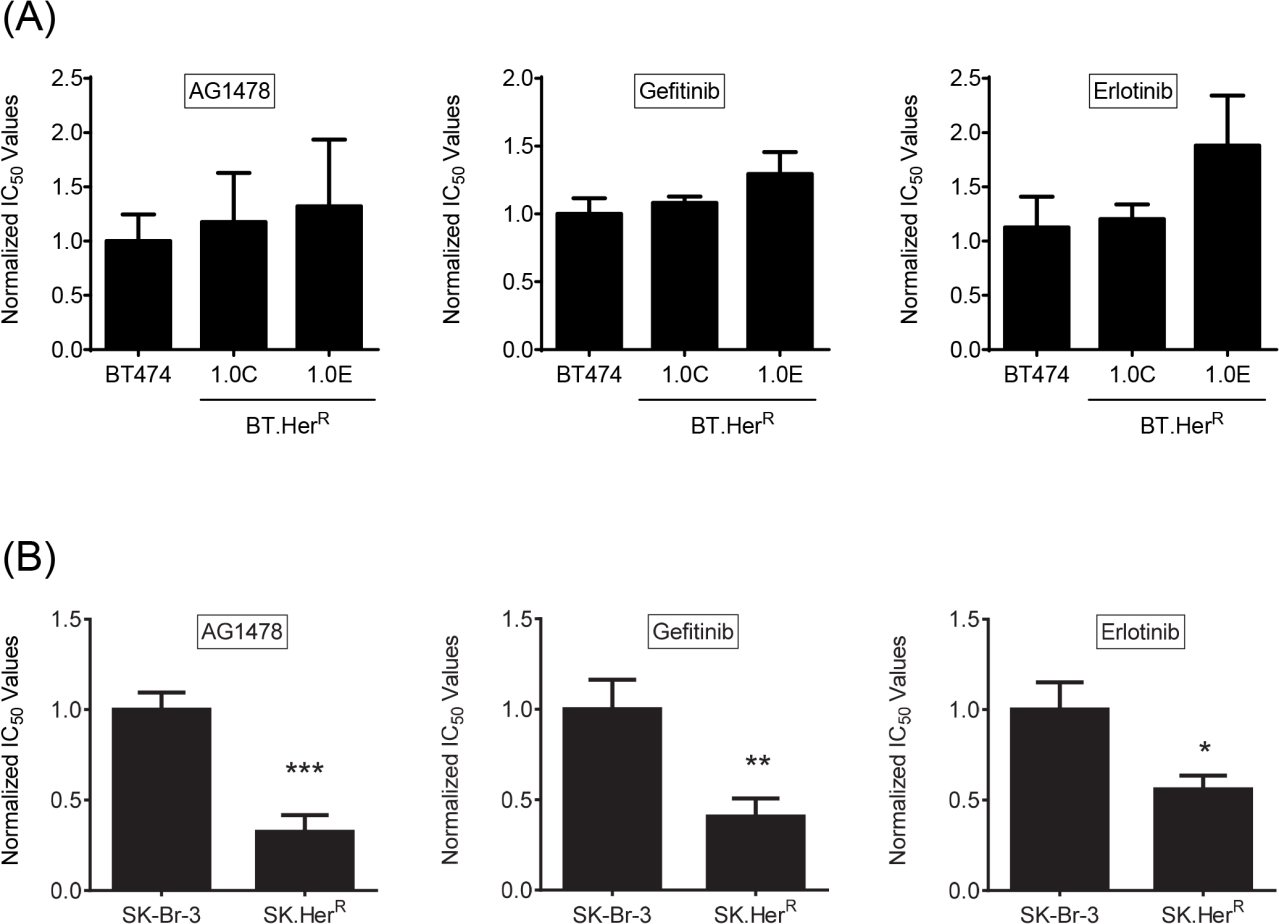
BT.Her^R^ and SK.Her^R^ cells demonstrate different sensitivities to EGFR inhibitors in the absence of trastuzumab. Cell proliferation in AG1478 (left), gefitinib (middle) and erlotinib (right) was measured by SRB assay after exposure to 0.1% DMSO or increasing concentrations of the respective drugs. IC_50_ values were obtained using CalcuSyn and normalized to the average IC_50_ for the appropriate parental cell line. Each bar represents the mean of three independent experiments ±SD. **(A)** Relative sensitivity for BT474 parental and two BT.Her^**R**^ clones (1.0C and 1.0E) treated with DMSO or drugs for 7 days. **(B)** Relative sensitivity for SK-Br-3 parental and SK.Her^**R**^ cells treated with DMSO or drugs for 5 days. *, p<0.05; **, p<0.01; ***, p<0.001 compared to parental cells.

We next wanted to determine if this same phenotype would be observed in other trastuzumab-resistant cell lines. We first compared the sensitivity of BT.Her^R^ and SK.Her^R^ cells to three EGFR-specific tyrosine kinase inhibitors—AG1478, gefitinib and erlotinib—in the absence of trastuzumab. There was no significant difference in cell proliferation between the parental BT474 and resistant BT.Her^R^ cell lines exposed to AG1478, gefitinib or erlotinib (Fig 1A), consistent with published data showing that enhanced sensitivity to EGFR inhibition in BT.Her^R^ cells requires the presence of trastuzumab. SK.Her^R^ cells, on the other hand, demonstrated a statistically significant two- to four-fold greater sensitivity to AG1478, gefitinib and erlotinib than parental SK-Br-3 cells, even in the absence of trastuzumab (Fig 1B), suggesting an inherent difference between BT.Her^R^ and SK.Her^R^ cells in their signaling through EGFR when Her2 is still active.

### t-Darpp over-expression confers sensitivity to EGFR inhibition

**Fig 2:**
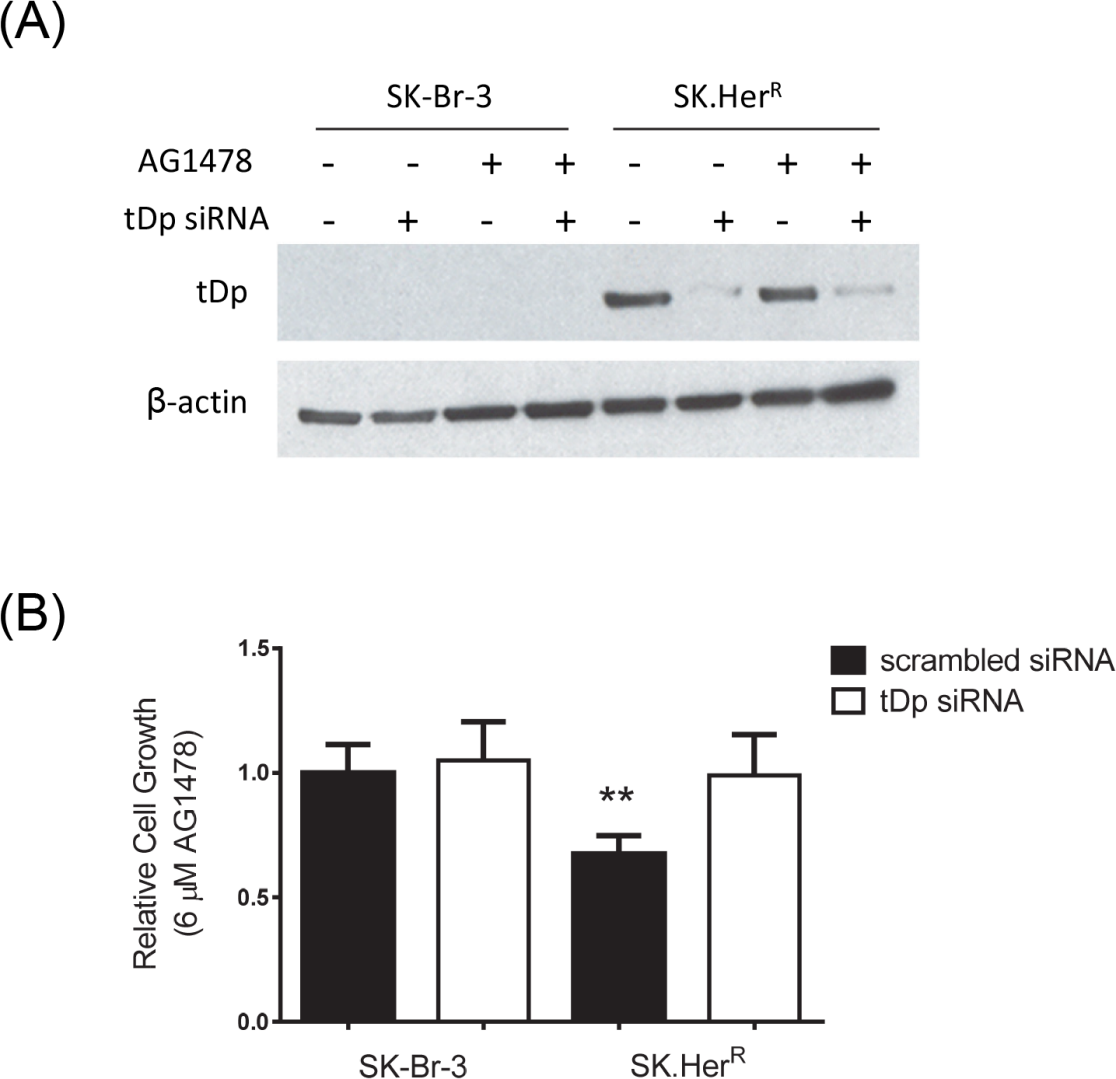
t-Darpp is responsible for increased EGFR sensitivity in SK.Her^R^ cells. SK-Br-3 and SK.Her^**R**^ cells were transfected with scrambled (-) or tDp siRNA (+). 24 hours later, cells were treated with DMSO or AG1478 for an additional 24 hours. **(A)** Western analysis to confirm siRNA knockdown. **(B)** SRB cell proliferation assay to determine relative sensitivity to AG1478. Shown are the averages (± S.D.) of four experiments, with normalization to DMSO-treated controls in all cases. **, p<0.01.

The difference in response to EGFR inhibitors between BT.Her^R^ and SK.Her^R^ cell lines led us to investigate the mechanism for the increased sensitivity in SK.Her^R^ cells. We and others have previously reported that over-expression of t-Darpp is sufficient to confer resistance to trastuzumab [17–19], so we wanted to determine if t-Darpp over-expression was responsible for the sensitization to EGFR inhibitors as well. Because SK.Her^R^ cells over-express endogenous t-Darpp, we first asked if t-Darpp was necessary for the enhanced sensitivity to EGFR inhibition in these cells. We performed siRNA knock-down of t-Darpp in SK.Her^R^ and SK-Br-3 cells and examined cell growth in 6μM AG1478 (the approximate IC_50_ for the parental cells), relative to cells treated with control siRNA. The sensitivity to EGFR inhibition in SK.Her^R^ cells was abrogated by t-Darpp knock-down (Fig 2), suggesting that t-Darpp is essential for increased sensitivity to EGFR inhibition.

To determine if t-Darpp was sufficient for conferring sensitivity to EGFR inhibition in cells that were not previously selected for trastuzumab resistance, we used SK.tDp cells stably transfected with t-Darpp cDNA and compared with SK.empty cells transfected with an empty vector (SK.empty). As an additional control, we used cells that had been transfected with both t-Darpp and Darpp-32 (SK.dDp cells). We have previously reported that SK.tDp cells are resistant to trastuzumab and SK.dDp cells have lost that resistance [17].

**Fig 3:**
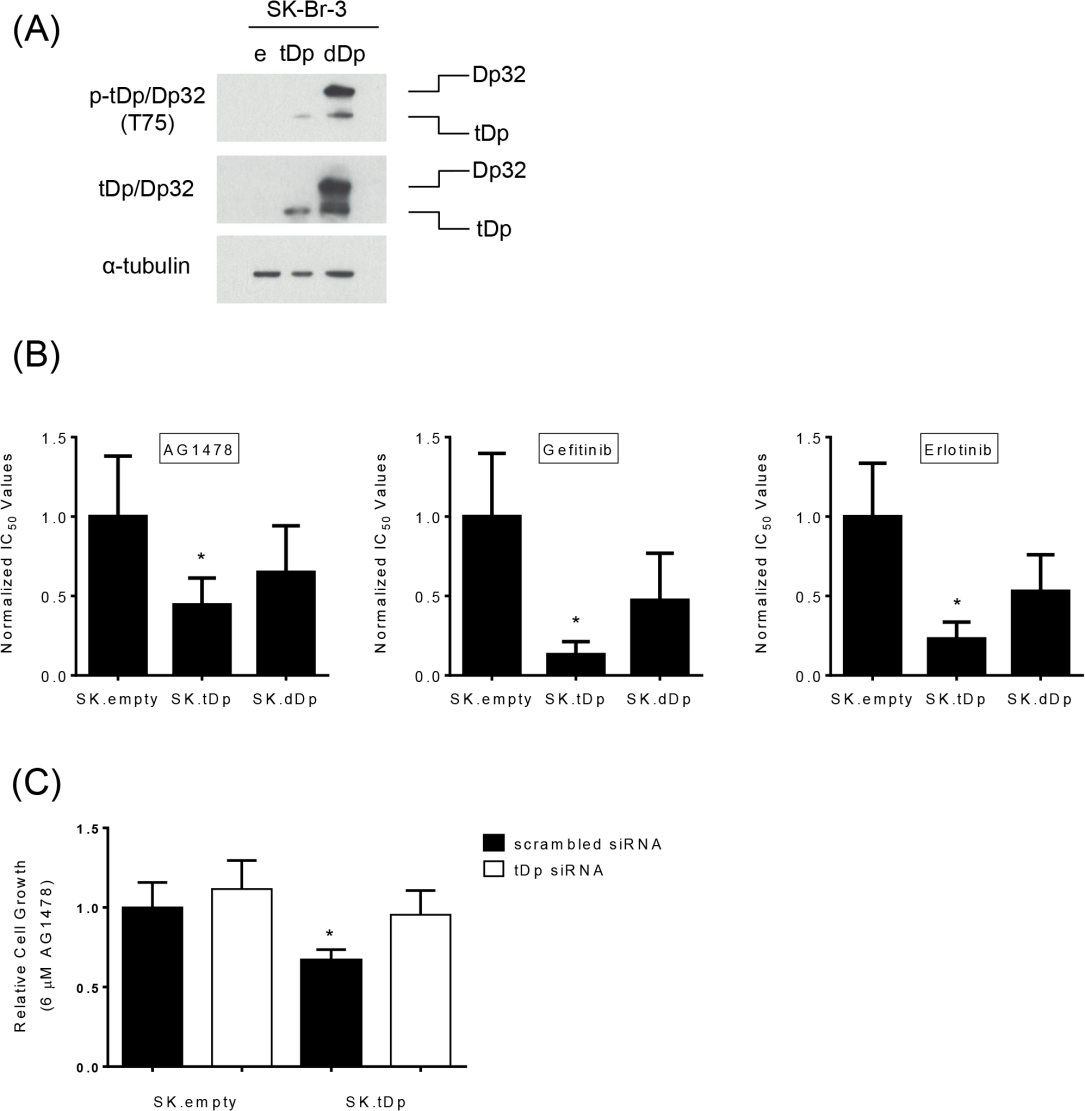
Increased sensitivity to EGFR inhibition in SK.tDp cells. **(A)** Western analysis showing relative amounts of Darpp-32 and t-Darpp in stably transfected SK-Br-3 cells: SK.empty (e), SK.tDp (tDp) and SK.flag-Dp32. tDp (dDp). **(B)** Normalized IC_50_ values for transfected SK-Br-3 cells treated with AG1478 (left), gefitinib (middle) or erlotinib (right). Each bar represents the mean of three independent experiments ± S.D. **(C)** SK.empty and SK.tDp cells were transfected with scrambled or tDp siRNA. After 24 hours, cells were treated with DMSO or AG1478 for an additional 48 hours and an SRB cell proliferation assay was performed to determine relative sensitivity to AG1478. Shown are averages (± S.D.) of three experiments normalized to DMSO-treated controls in all cases. Error bars indicate SD. *, p<0.05.

We first confirmed that the over-expression of t-Darpp alone caused increased cell proliferation and resistance to trastuzumab in the current study and that knock-down of t-Darpp by siRNA reversed these effects in SK.tDp cells (S2 Fig). We next evaluated the sensitivity of SK.tDp, SK.empty and SK.dDp cells to EGFR inhibition. In SRB cell proliferation assays, SK.tDp cells were 3- to 5-fold more sensitive than SK.empty cells to AG1478, gefitinib and erlotinib, whereas SK.dDp cells demonstrated an intermediate sensitivity that did not reach statistical significance (Fig 3B). Colony formation assays using 6μM AG1478 also indicated that SK.tDp cells were more sensitive to AG1478 compared to SK.empty cells (S3 Fig). Moreover, siRNA knock-down of t-Darpp in SK.tDp cells reversed the sensitivity to AG1478 (Fig 3C). Taken together, these results demonstrate that t-Darpp is sufficient to confer sensitivity to EGFR inhibition in SK-Br-3 cells and they extend previous findings that Darpp-32 over-expression reverses the t-Darpp phenotype [17].

### Dual EGFR and HER2 inhibition is synergistic in cells over-expressing t-Darpp

Given the enhanced sensitivity of SK.Her^R^ and SK.tDp cells to EGFR inhibition, we wanted to determine if trastuzumab and EGFR inhibitors would have synergistic effects on these cells. We used combination index analysis to study the interaction of trastuzumab with AG1478 and erlotinib in SK.empty and SK.tDp cell lines. The AG1478 + trastuzumab combination was highly synergistic in SK.tDp cells but only additive in SK.empty cells (Table 1). The combination of erlotinib + trastuzumab was highly synergistic in SK.tDp cells (ED_50_ of 0.39 ± 0.10, ED_75_ of 0.50 ± 0.10) and moderately synergistic in SK.empty cells (ED_50_ of 0.56 ± 0.12, ED_75_ of 0.72 ± 0.12) (Table 1). Taken together, these results suggest that t-Darpp over-expression can, at a minimum, enhances the synergistic effect between Her2 and EGFR inhibitors.

**Table 1:** Combination Index.

Combination Index^1^				
Cell Line	AG1478 + Trastuzumab		Erlotinib + Trastuzumab	
	ED_50_ ^2^	ED_75_ ^2^	ED_50_	ED_75_
SK.empty	1.00 ± 0.14	1.21 ± 0.09	0.56 ± 0.12	0.72 ± 0.12
SK.tDp	0.39 ± 0.10**	0.62 ± 0.05***	0.39 ± 0.10	0.50 ± 0.10

^1^Combination Index values indicate the degree of synergy (CI <0.9), additivity (CI 0.9–1.1) or antagonism (CI >1.1) for two drugs maintained in a fixed ratio.

^2^ED_50_ and ED_75_ are the effective doses at which 50% and 75% of cell killing occurred, respectively.

**, p<0.01

***, p<0.005

### T75 residue of t-Darpp may be responsible for conferring increased EGFR sensitivity

**Fig 4:**
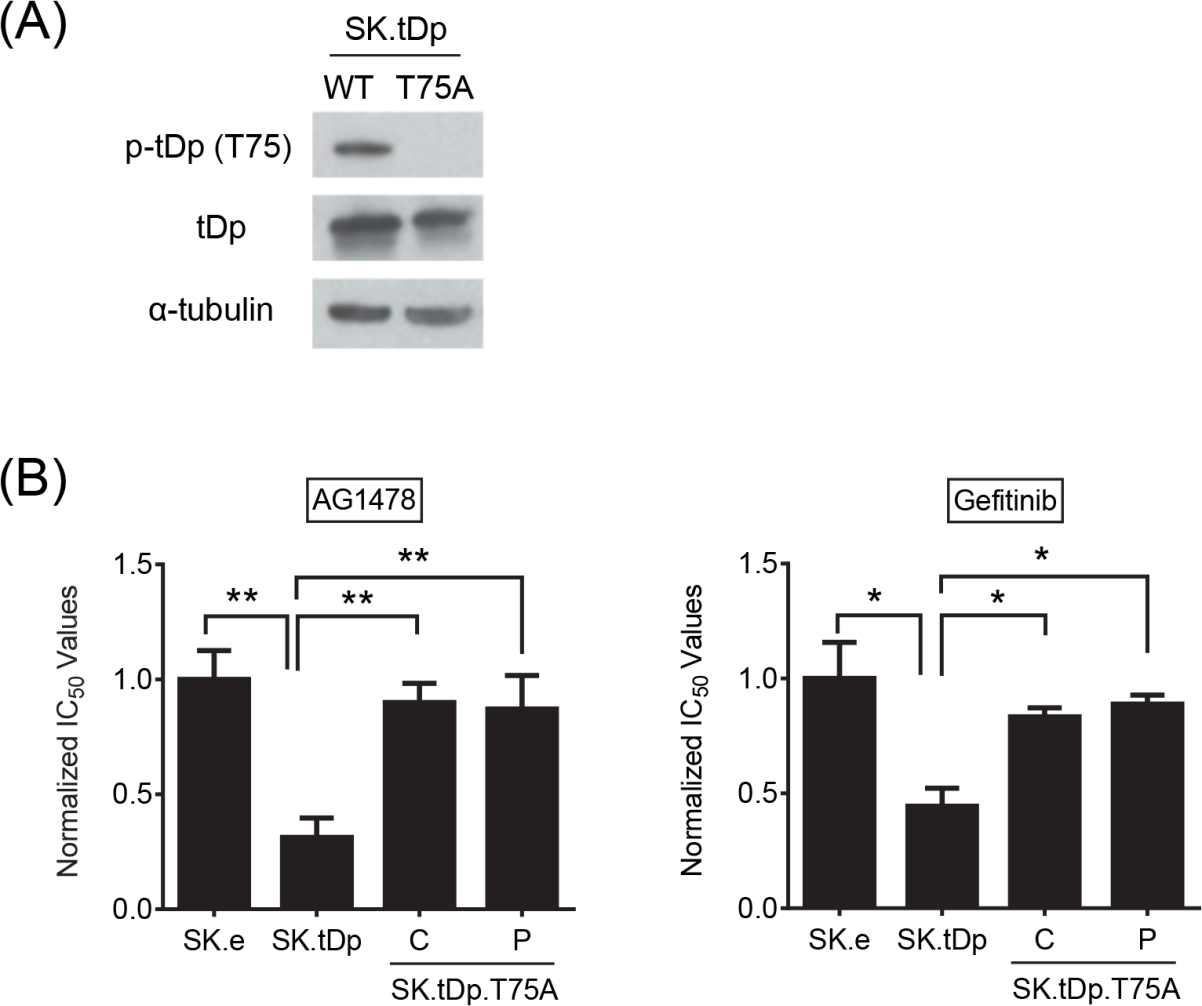
T75 residue of t-Darpp might be responsible for conferring increased EGFR sensitivity. **(A)** Western analysis showing relative amounts of p-t-Darpp (p-T75) and t-Darpp (tDp) in stably transfected SK-Br-3 cells. α–tubulin was used as a loading control. **(B)** Normalized IC_50_ values for transfected SK-Br-3 cells treated with AG1478 (left) or gefitinib (right). A clone (C) and a pooled population (P) are shown for the SK.tDp-T75A cells. Each bar represents the mean (±S.D.) IC_50_ of three independent experiments, with each normalized to the average IC_50_ of the SK.empty cells. *, p<0.05; **, p<0.01.

Although t-Darpp regulation has yet to be studied in-depth, phosphorylation at an internal threonine residue (T75, using the numbering scheme of Darpp-32) appears to be required for t-Darpp to confer trastuzumab resistance [19]. This same T75 site is important for regulating Darpp-32’s function as a PKA inhibitor [22]. To determine if phosphorylation at T75 is required for t-Darpp’s effects on EGFR, we stably transfected SK-Br-3 cells with t-Darpp cDNA encoding an alanine substitution at residue T75 (SK.tDp-T75A) and confirmed that these cells expressed t-Darpp lacking T75 phosphorylation (Fig 4A). In cell proliferation assays, SK.tDp cells again exhibited a 3-fold increased sensitivity to AG1478, relative to SK.empty controls, but SK.tDp-T75A cells were not more sensitive to AG1478 than parental cells (Fig 4B). Similar results were obtained with gefitinib (Fig 4B). These results suggest that the T75 residue might play an important role in t-Darpp’s ability to confer EGFR sensitivity.

### EGFR signaling is enhanced in SK.Her^R^ and SK.tDp cells

**Fig 5:**
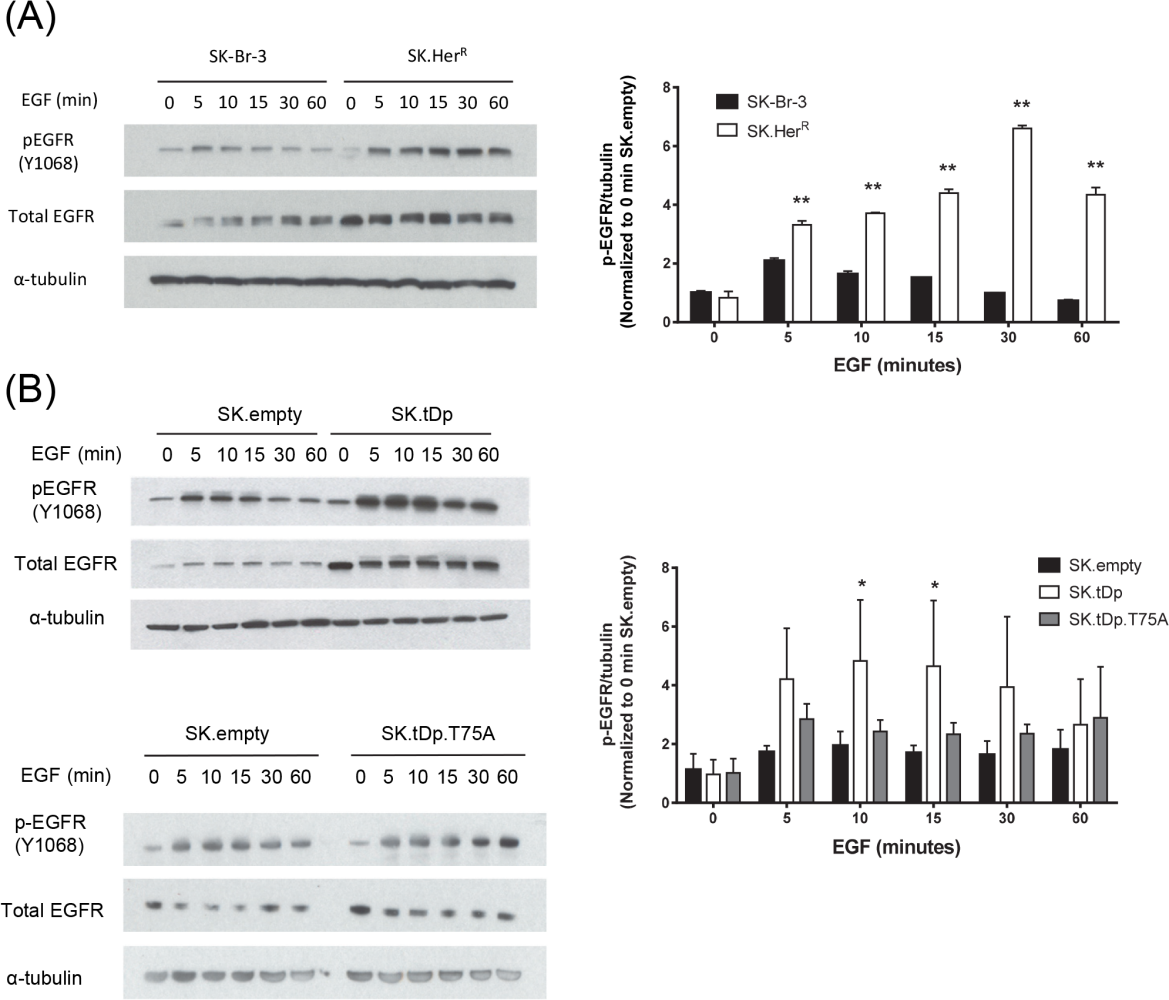
Increased EGFR activation in cells over-expressing t-Darpp. Western analysis (left) and quantification (right) of cells serum-starved overnight (16 hours) followed by 100 ng/ml EGF stimulation for the indicated time points. α–tubulin was used as a loading control. Protein expression was quantified using the ImageJ software. p-EGFR data was normalized to α–tubulin in each lane and represented as the fold change relative to the zero time point p-EGFR signal for the SK-Br-3 or SK.empty cell line. **(A)** SK-Br-3 and SK.Her^**R**^ cells **(B)** SK.empty, SK.tDp and SK.tDp-T75A cells. *, p <0.05; **, p<0.01.

To investigate the possible role of EGFR activation in modulating the increased sensitivity to EGFR inhibition, we measured total EGFR levels and phosphorylation at tyrosine 1068 (Y1068) in serum-starved cells over the course of one hour of EGF stimulation (Fig 5). Both SK.Her^R^ and SK.tDp cells had elevated EGFR protein, relative to their respective controls, even in the absence of EGF, and this remained steady over the course of EGF stimulation. We observed a significantly more dramatic increase in phospho-EGFR levels after the addition of EGF in both SK.Her^R^ and SK.tDp cells, compared with their respective control cell lines. SK.tDp-T75A cells, on the other hand, did not have significantly elevated EGFR nor p-EGFR levels, compared to SK.empty controls, in the absence or presence of EGF (Fig 5B). These results suggest that t-Darpp promotes enhanced signaling through EGFR and that phosphorylation at the T75 residue may be required for this effect.

To examine further the observation of higher steady-state protein levels in the presence of wild type t-Darpp, we measured EGFR protein stability using cycloheximide. The average half-life of EGFR in SK.tDp cells was 42 ± 11 hours compared to 20 ± 5 hours in the SK.empty cells (p = 0.032). The average half-life in SK.Her^R^ cells was 26 ± 7 hours compared to that in parental SK-Br-3 cells of 11 ± 3 hours (p = 0.023). A representative gel analysis and quantification of all cycloheximide experiments are shown in S4 Fig. These results support the idea that t-Darpp over-expression enhances signaling through EGFR, either by stabilizing the protein or by promoting its stimulation, and thereby sensitizes cells to EGFR inhibition.

## Discussion

Although trastuzumab is useful in treating patients with HER2^+^ breast cancer, overcoming trastuzumab resistance remains an important challenge to improve patient outcomes. The mechanisms of acquired resistance are still being determined, but the upregulation of other HER family receptors as a compensatory signaling mechanism and sustained signaling through the PI3K/Akt pathway are well established components of resistance [27]. The combination of trastuzumab with EGFR inhibitors represents a viable strategy to address this resistance mechanism.

We demonstrate that t-Darpp over-expression sensitizes SK.Her^R^, but not BT.Her^R^ cells, to EGFR inhibition when trastuzumab itself is not present. Although both cell lines have high HER2 expression levels, it is well accepted that BT474 and SK-Br-3 cells have different signaling patterns. The difference that may be most relevant to the current study is the inherent difference in receptor dimerization patterns between the two cell lines, with BT474 cells signaling primarily through HER2 homodimers and SK-Br-3 cells signaling through HER2 heterodimerization with EGFR and possibly HER3 [28]. We reported previously that BT.Her^R^ are sensitized to AG1478 only in the presence of trastuzumab, which is consistent with a model in which EGFR can only compensate for HER2 signaling when HER2 homodimers are down-regulated by trastuzumab [28]. In SK.Her^R^ cells, on the other hand, the greater reliance on EGFR as part of the normal signaling mechanism, coupled with enhanced EGFR levels, stability and/or activity in those cells (Fig 5 and S4 Fig), most likely leads to greater sensitivity to EGFR inhibition even in the absence of HER2 inhibition by trastuzumab (Fig 1). The fact that the same phenotype was observed in SK.tDp cells, was reversed by Darpp-32 (Fig 3), and was not observed in SK.tDp-T75A cells (Fig 4), suggests that the enhancing effect is mediated by t-Darpp itself and that T75 phosphorylation is required for t-Darpp to confer this effect.

Another reported signaling discrepancy is that BT474 cells express estrogen receptor (ER), whereas SK-Br-3 cells do not. Wang et al. recently found that upon sustained HER2 inhibition with trastuzumab, ER acts as a crucial and compensatory survival pathway in HER2^+^ and ER^+^ cancers [29]. Although we did not investigate changes in estrogen receptor signaling in trastuzumab resistance BT474 cells, it is possible that BT.Her^R^ cells do not shift to EGFR signaling as a primary pathway (in the absence of trastuzumab) because they have the ER pathway available as a more robust back-up than EGFR. SK.Her^R^ cells, on the other hand, which lack ER, might be more likely to upregulate other HER family members as a mechanism of resistance because they lack other significant signaling pathways.

The EGFR sensitizing effect also translates into greater synergy between EGFR inhibitors and trastuzumab in SK.Her^R^ and SK.tDp cells compared with parental cells. An additivity or moderate synergy in parental cells is consistent with published reports demonstrating that gefitinib in combination with trastuzumab produces a small synergistic or additive effect in SK-Br-3 cells [30] and is perhaps reflective of the important role that EGFR plays in the normal signaling phenotype of these cells. The greater synergy in SK.Her^R^ and SK.tDp cells might be explained by inhibition of stabilized HER2/EGFR heterodimers or combined inhibition of HER2/EGFR and EGFR/EGFR or EGFR/HER3 dimers that compensate for HER2/EGFR in SK.Her^R^ and SK.tDp cells. Further investigation of receptor dimerization patterns in SK.Her^R^ and SK.tDp cells could reveal important details about the role of dimerization in determining drug sensitivities in cell lines and clinical cases.

The mechanism by which t-Darpp over-expression promotes enhanced sensitivity to EGFR inhibition and synergism with HER2 inhibition remains unclear. We showed here that t-Darpp expression resulted in a more robust activation of the EGFR pathway, possibly by stabilizing EGFR protein and, by extension, the dimers in which it participates. El-Rifai et al. previously reported that t-Darpp can bind HER2 in trastuzumab-resistant HR5 cells, a BT474-derived cell line, in a complex with HSP90 [18]. The same group also reported that t-Darpp can bind and stabilize HER2 in a complex to inhibit trastuzumab from correctly binding with the HER2 receptor in esophageal cells [20]. We have not yet detected a direct interaction between t-Darpp and EGFR (G. Lenz, N. Brink-Goodman, E. Denny, unpublished observations), but additional experiments are needed to determine if t-Darpp confers its effects through such a physical interaction with EGFR or perhaps via HER2.

There might be a more mechanistic effect of t-Darpp on EGFR signaling, not just through formation or stabilization of complexes. This is evidenced by the lack of sensitization to EGFR inhibition and the lack of enhanced p-EGFR stimulation in SK.tDp-T75A cells. The T75 phosphorylation site is crucial for the full-length protein, Darpp-32, to modulate PKA signaling and Hamel et al. demonstrated that the T75A mutation in t-Darpp abolishes its ability to confer trastuzumab resistance in BT474 cells [19]. It is possible that T75 phosphorylation is required for physical interaction with EGFR and that this promotes enhanced EGFR signaling and trastuzumab resistance, but it is also possible that t-Darpp’s effect is manifested via an effect on PKA signaling, consistent with a model that we have previously proposed [17]. We are currently investigating the regulation of t-Darpp by T75 phosphorylation as well as the importance of this site to t-Darpp’s effects on PKA.

Our results reiterate the importance of molecular profiling to determine the most effective therapies. Pre-screening of EGFR status is frequently performed in lung and gastric cancers to determine the effectiveness of EGFR-targeted therapies including gefitinib and erlotinib. Our results suggest that t-Darpp over-expression in combination with EGFR activation and dimerization patterns might correlate with sensitivity to EGFR inhibition in trastuzumab-resistant tumors, and t-Darpp itself could potentially be pursued as a new molecular target. These findings may be indicative of a novel function for t-Darpp in trastuzumab-resistant breast cancers. Further studies are necessary to delineate the mechanism by which t-Darpp causes this effect.

## Supporting Information

S1 Fig t-Darpp and Darpp-32 in parental and trastuzumab-resistant cell lines. Phosphorylated t-Darpp and total t-Darpp levels were measured by Western analysis in a panel of breast cancer cell lines. BT 1.0C and BT 1.0E are trastuzumab resistant BT474 (BT.Her^R^) cells that were continuously selected in the presence of trastuzumab. β-actin was used as a protein loading control.
(PDF)

S2 Fig Increased proliferation in SK.tDp cells in response to trastuzumab. SK.empty and SK.tDp cells were transfected with scrambled or tDp siRNA for 24 hours and subsequently treated with DMSO or trastuzumab for an additional 24 hours. A SRB cell proliferation assay was used to determine relative sensitivity to trastuzumab. Shown are the averages ± SD of three experiments normalized to DMSO-treated controls. **, p<0.01; *, p<0.05.
(PDF)

S3 Fig SK.tDp cells demonstrate increased sensitivity to EGFR inhibition. Colony formation assay showing percent proliferation at various concentrations of AG1478. Colonies, defined as having at least 50 cells, were counted after 14 days treatment.
(PDF)

S4 Fig Presence of t-Darpp stabilizes EGFR protein levels. Western analysis (left) and quantification (right) showing **(A)** SK.empty and SK.tDp cells and **(B)** SK-Br-3 and SK.Her^R^ cells treated with 80 μg/ml cycloheximide (CHX) over 72 or 48 hours, respectively. α–tubulin was used as a loading control. EGFR protein expression was normalized to α–tubulin and represented as the fold change relative to the untreated control for each cell line. Error bars represent the SD in three independent experiments.
(PDF)

S1 File Raw data for Figs 1–5. Each tab in the spreadsheet contains raw data for an individual figure, as labeled. Data for Fig 1A and 1B are calculated IC_50_ values for the indicated cell lines and drugs, from three replicate experiments. Data for Fig 2B are average (±S.D.) cell proliferation read-outs from each of four experiments, each done in quadruplicate. Data for Fig 3B and 3C are calculated IC_50_ values and cell proliferation read-outs, respectively, for the indicated transfected cells lines. Data for Fig 4B are calculated IC_50_ values for the indicated transfected cells (wild type and mutant t-Darpp) from three replicate experiments. Data for Fig 5A and 5B are individual ImageJ readings of phospho-EGFR and tubulin band intensities in the indicated cell lines and EGF incubation times, taken from two to seven gel images.
(XLS)

S1 Table Combination index analysis of BT474 and BT.Her^R^ cell lines. Combination index analysis showing the interaction of trastuzumab with EGFR-specific kinase inhibitors AG1478 and erlotinib in parental and trastuzumab-resistant BT.Her^R^ cell lines. The combination of either AG1478 or erlotinib with trastuzumab produced high levels of synergy in all cell lines.
(PDF)
