# Supplementary figures and images for: t-Darpp Promotes Enhanced EGFR Activation and New Drug Synergies in Her2-Positive Breast Cancer Cells
Source: PLoS One. 2015 Jun 29;10(6):e0132267. doi: 10.1371/journal.pone.0132267 (PMC4488293; doi:10.1371/journal.pone.0132267)

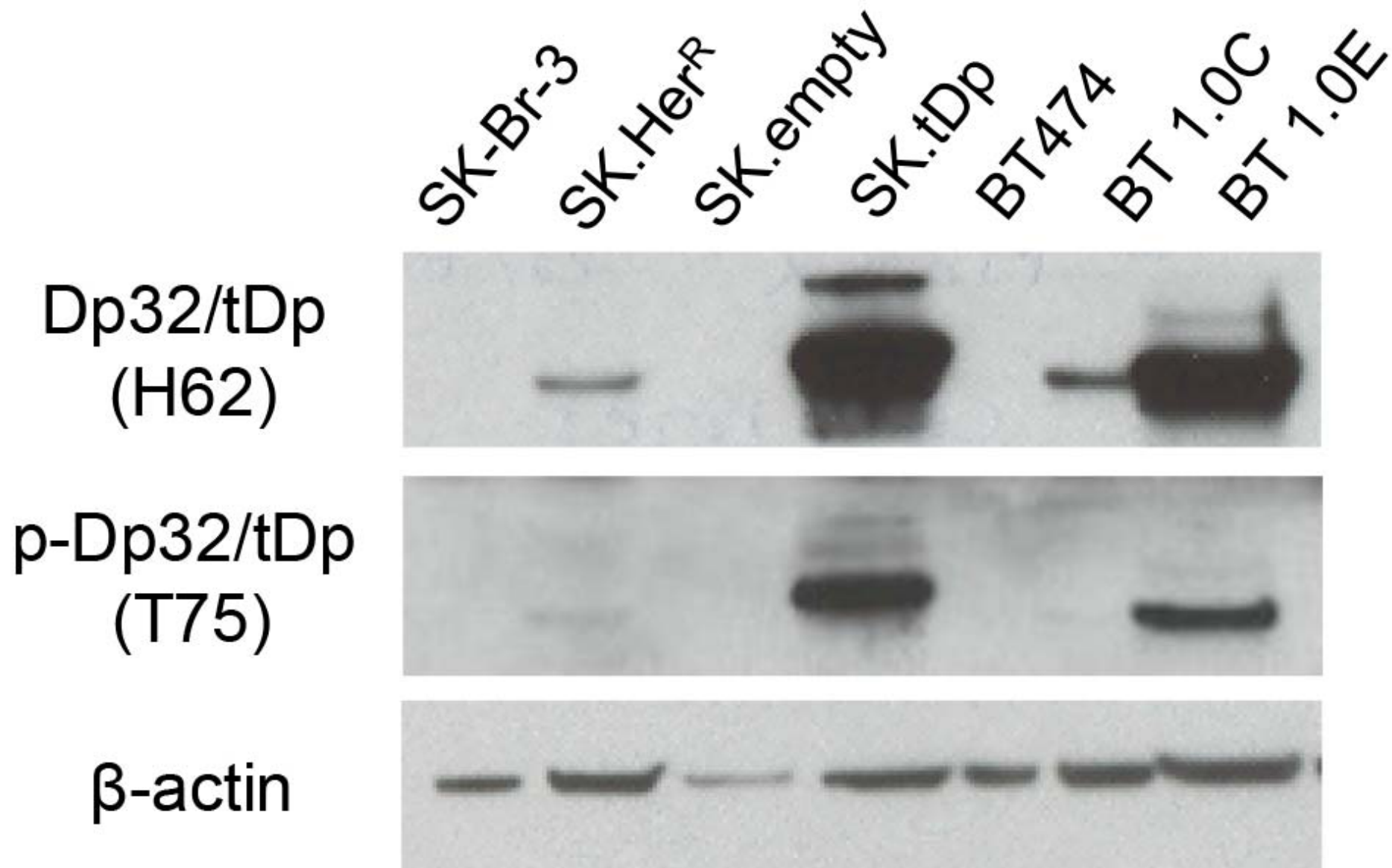

Supplement: S1 Fig — Phosphorylated t-Darpp and total t-Darpp levels were measured by Western analysis in a panel of breast cancer cell lines. BT 1.0C and BT 1.0E are trastuzumab resistant BT474 (BT.HerR) cells that were continuously selected in the presence of trastuzumab. β-actin was used as a protein loading control. (PDF) [file pone.0132267.s001.pdf]

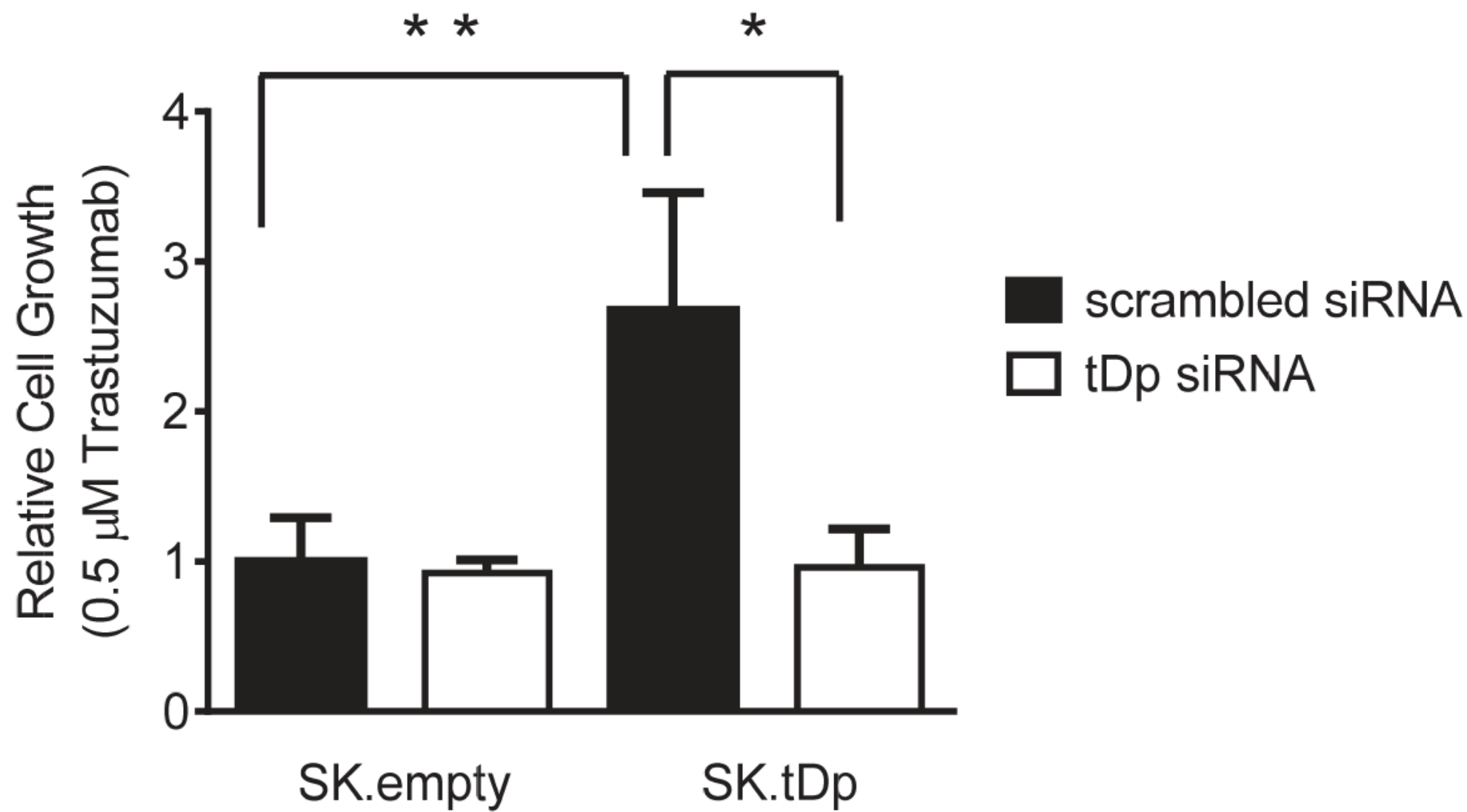

Supplement: S2 Fig — SK.empty and SK.tDp cells were transfected with scrambled or tDp siRNA for 24 hours and subsequently treated with DMSO or trastuzumab for an additional 24 hours. A SRB cell proliferation assay was used to determine relative sensitivity to trastuzumab. Shown are the averages ± SD of three experiments normalized to DMSO-treated controls. **, p<0.01; *, p<0.05. (PDF) [file pone.0132267.s002.pdf]

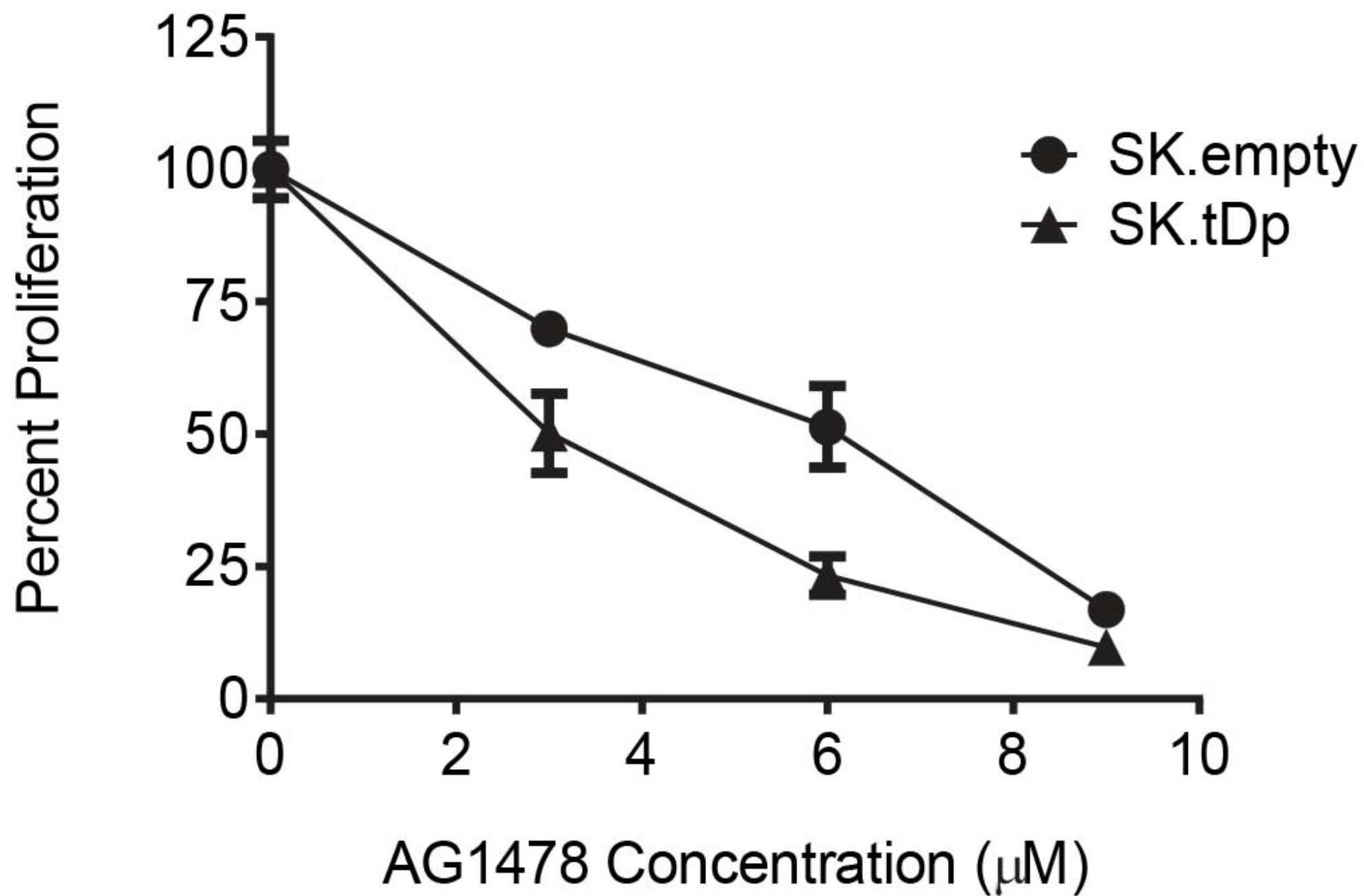

Supplement: S3 Fig — Colony formation assay showing percent proliferation at various concentrations of AG1478. Colonies, defined as having at least 50 cells, were counted after 14 days treatment. (PDF) [file pone.0132267.s003.pdf]

(A)

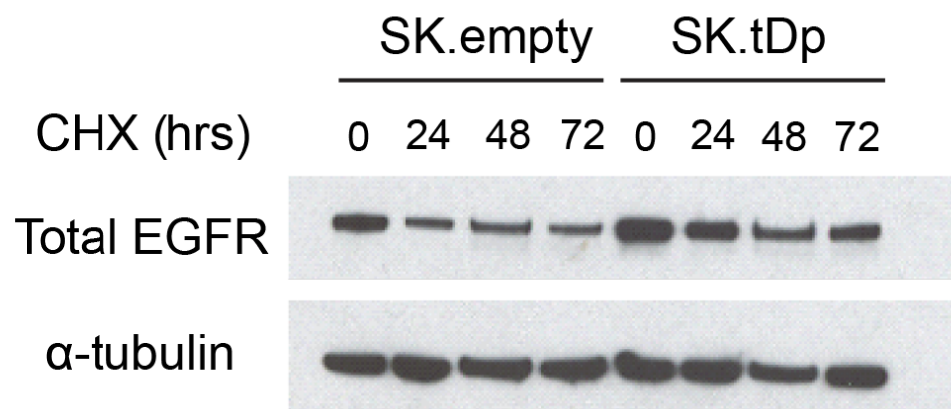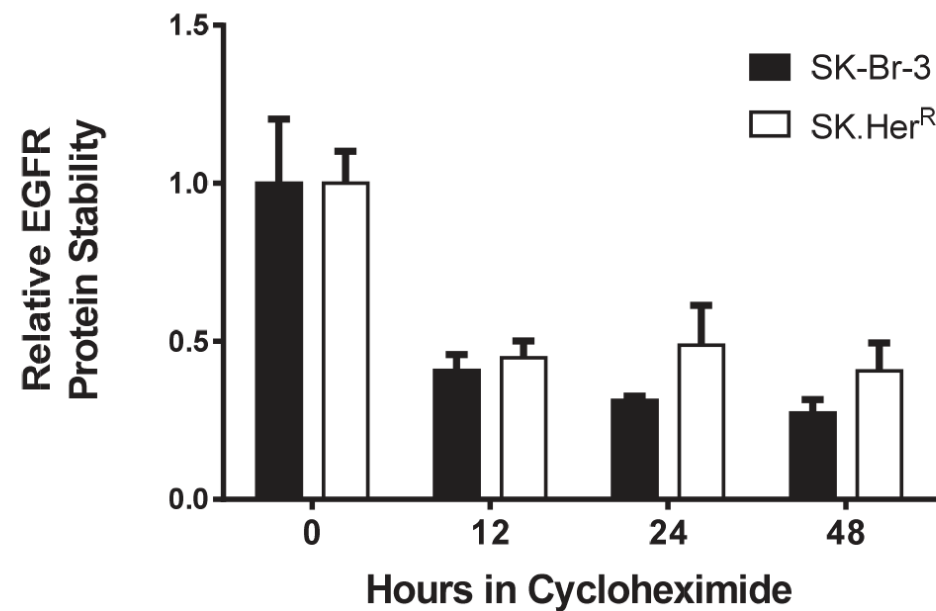

(B)

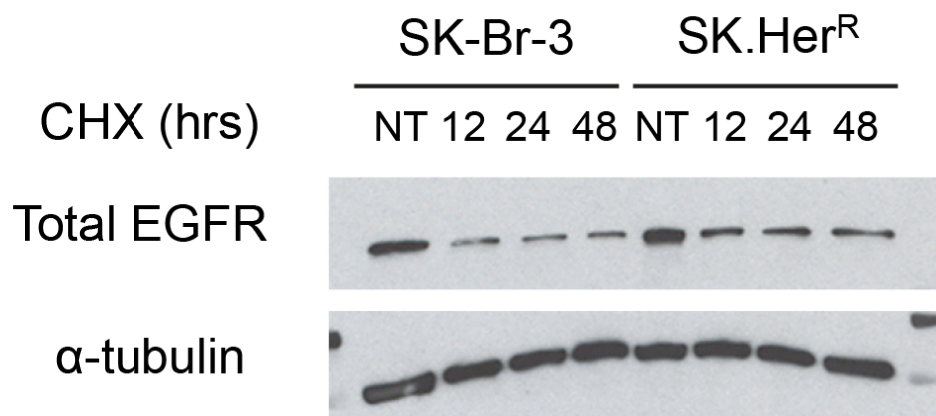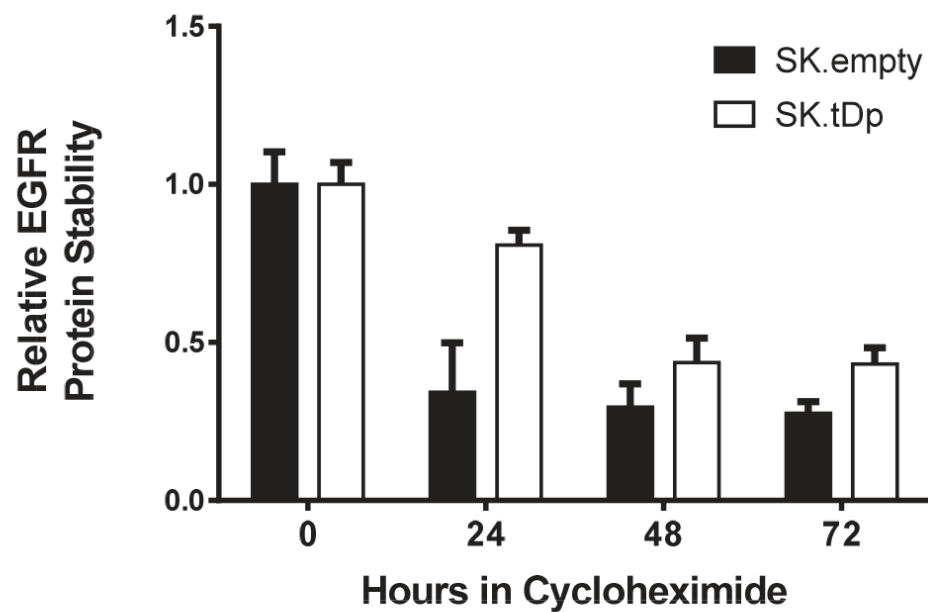

Supplement: S4 Fig — Western analysis (left) and quantification (right) showing (A) SK.empty and SK.tDp cells and (B) SK-Br-3 and SK.HerR cells treated with 80 μg/ml cycloheximide (CHX) over 72 or 48 hours, respectively. α–tubulin was used as a loading control. EGFR protein expression was normalized to α–tubulin and represented as the fold change relative to the untreated control for each cell line. Error bars represent the SD in three independent experiments. (PDF) [file pone.0132267.s004.pdf]
