# Supplementary material for: t-Darpp Promotes Enhanced EGFR Activation and New Drug Synergies in Her2-Positive Breast Cancer Cells
Source: PLoS One. 2015 Jun 29;10(6):e0132267. doi: 10.1371/journal.pone.0132267 (PMC4488293; doi:10.1371/journal.pone.0132267)
Supplement: S1 Table — Combination index analysis showing the interaction of trastuzumab with EGFR-specific kinase inhibitors AG1478 and erlotinib in parental and trastuzumab-resistant BT.HerR cell lines. The combination of either AG1478 or erlotinib with trastuzumab produced high levels of synergy in all cell lines. (PDF) [file pone.0132267.s006.pdf]

**Supplementary Table 1. Combination Index[1]**				
Cell Line	AG1478 + Trastuzumab		Erlotinib + Trastuzumab	
	ED_{50}[2]	ED_{75}[2]	ED_{50}	ED_{75}
BT474	0.25 ± 0.12	0.66 ± 0.22	0.60 ± 0.23	0.40 ± 0.14
BT.Her^R 1.0C	0.48 ± 0.09	0.65 ± 0.09	0.31 ± 0.17	0.27 ± 0.17
BT.Her^R 1.0E	0.31 ± 0.09	0.70 ± 0.22	0.26 ± 0.03	0.32 ± 0.08

[1]Combination Index values indicate the degree of synergy (CI <0.9), additivity (CI 0.9-1.1) or antagonism (CI >1.1) for two drugs maintained in a fixed ratio.

[2]ED_{50} and ED_{75} are the effective doses at which 50% and 75% of cell killing occurred, respectively.
